# Population Genomic Analysis Reveals a Rich Speciation and Demographic History of Orang-utans (*Pongo pygmaeus* and *Pongo abelii*)

**DOI:** 10.1371/journal.pone.0077175

**Published:** 2013-10-23

**Authors:** Xin Ma, Joanna L. Kelley, Kirsten Eilertson, Shaila Musharoff, Jeremiah D. Degenhardt, André L. Martins, Tomas Vinar, Carolin Kosiol, Adam Siepel, Ryan N. Gutenkunst, Carlos D. Bustamante

**Affiliations:** 1 Department of Statistics, Stanford University, Stanford, California, United States of America; 2 Department of Genetics, Stanford University, Stanford, California, United States of America; 3 Bioinformatics Core, Gladstone Institutes, San Francisco, California, United States of America; 4 Department of Biological Statistics and Computational Biology, Cornell University, Ithaca, New York, United States of America; 5 Department of Molecular and Cellular Biology, University of Arizona, Tucson, Arizona, United States of America; 6 Department of Applied Informatics, Comenius University, Bratislava, Slovakia; 7 Institute of Population Genetics, Vetmeduni Vienna, Vienna, Austria; Erasmus University Medical Center, The Netherlands

## Abstract

To gain insights into evolutionary forces that have shaped the history of Bornean and Sumatran populations of orang-utans, we compare patterns of variation across more than 11 million single nucleotide polymorphisms found by previous mitochondrial and autosomal genome sequencing of 10 wild-caught orang-utans. Our analysis of the mitochondrial data yields a far more ancient split time between the two populations (∼3.4 million years ago) than estimates based on autosomal data (0.4 million years ago), suggesting a complex speciation process with moderate levels of primarily male migration. We find that the distribution of selection coefficients consistent with the observed frequency spectrum of autosomal non-synonymous polymorphisms in orang-utans is similar to the distribution in humans. Our analysis indicates that 35% of genes have evolved under detectable negative selection. Overall, our findings suggest that purifying natural selection, genetic drift, and a complex demographic history are the dominant drivers of genome evolution for the two orang-utan populations.

## Introduction

*Pongo pygmaeus* and *Pongo abelii*, Bornean and Sumatran orang-utans, respectively, are arboreal Asian great apes whose distributions are exclusive to the islands of Borneo and Sumatra. Here, we aim to assess the role of competing evolutionary forces including natural selection, genetic drift, and migration in shaping genome-wide patterns of variation cross 10 previously sequenced wild orang-utan genomes. Multiple factors have impacted the recent evolutionary history and current distributions of orang-utans, and it is important to understand these abiotic and biotic factors before turning to genome analysis. Three factors that are particularly important for understanding orang-utan genome variation are the biogeography of the region over the last million years, the social structure of orang-utans, and the impact of humans on orang-utan populations.

First, climatic and geographic changes during the middle to late Pleistocene greatly impacted the distribution of available habitat and interconnectivity of forests. Recurring glacial periods led to a cooler, drier and more seasonal climate [1] and contracted rain forest likely isolated populations of orang-utans. At the same time, the sea level dropped and continental shelf was exposed, forming land bridges among islands [1], [2] and creating opportunities for migration among previously isolated population or species [3]. The degree to which recurrent glacial periods may have been an isolating versus a connecting factor remains poorly understood. Rivers and mountain ridges further serve as genetic dispersal barriers within islands, as supported by multiple genetic studies [4]–[6]. Volcanic eruptions, especially the Toba super-eruption, could have also played a large role in patterning Sumatran orang-utan genome diversity as shown by Nater et al. [7], [8].

Second, the social structure of adult orang-utans is quite different from that of other diurnal primates and may play a dominant role in shaping patterns of genome variation. Orang-utans are the least gregarious great apes and their social structure can be described as semi-solitary [9]. The social structure is characterized by loosely organized groups of solitary females (and their young) who are often related to one another [10], [11]. Orang-utans exhibit sex-biased dispersal; males typically leave the natal area while females are more philopatric [10]–[14]. Resident females demonstrate a strong preference for mating with dominant (flanged) males [15]. This sex biased dispersal and potential reproductive skew will differentially impact the autosomes versus the sex chromosomes and mitochondrial DNA (mtDNA). The degree to which social organization has impacted the genetic structure of the two populations is under studied and few genome-wide analyses have addressed this issue.

Third, hunting of orang-utans by ancient hunter-gathers in Southeast Asia (∼40 kya) [16], hunting and habitat destruction from early farmers (∼4–5 kya) [17], [18], and recent human activities in the last two centuries (hunting, illegal pet trade and deforestation) [9], [19] have all led to dramatic population size decreases. The widespread deforestation on Borneo and Sumatra is rapidly limiting the habitat and resources available to orang-utans [20]. However, the degree to which human activity has reduced genetic variation in the two species remains uncharacterized at a genome-wide scale.

Therefore, a detailed study of population structure, demographic history, and natural selection may provide insight into orang-utan population history and evolution.

Estimates of split time of the two orang-utan populations vary greatly across different studies. Earlier studies based on the hypervariable control region I of mitochondrial DNA suggested a split time of 1.1–3.6 million years ago (mya) [5], [6], [21]. In contrast, two recent analyses on whole genome nuclear DNA sequences using methods based on single nucleotide polymorphisms (SNP) frequencies [22] or a coalescent hidden Markov model [23] estimated the split time to be 0.3–0.4 mya. These estimates are difficult to reconcile, because the studies are based on different data (mtDNA vs nuclear data) from different samples. Here we analyze SNPs found by previous direct genome sequencing of mtDNA and nuclear DNA from 10 individuals in the orang-utan genome project [22]. The combined analysis of complete autosomal data and mtDNA data from the same individuals, along with previously published mtDNA data from an independent set of samples [5], [6] gives us greater insight into organg-utan speciation history.

Orang-utans are the only Asian great ape, and although they represent the first hominid divergence, they share several convergent traits with humans [24] and provide an important model for human morphological evolution [25]. In the orang-utan genome paper [22], we provided a detailed study of genes under positive selection. However, the methodology we used in that paper relied on branch-site likelihood ratio tests [26], [27], which only utilized among-species divergence information and focused on only positive selection. In this study, we quantify the impact of natural selection on genetic variation in orang-utans by combining within-species polymorphism and between-species divergence data. We classify SNPs into functional categories and carry out a detailed analysis of their site-frequency spectra (SFS) to quantify potential differences in selective pressures between the Bornean and Sumatran populations. A modification of our previously published diffusion-based inference strategy [28] allows us to infer the strength of purifying selection on protein-coding genes while controlling for demographic history. To further understand the distribution of selective effects among different categories of functional mutations, we use predictions based on the PolyPhen-2 algorithm to classify amino acid changes as putatively benign, possibly damaging, and probably damaging [29]. To identify neutrally and non-neutrally evolving loci, we also undertake a genome-wide scan for genes under selection using a novel inference method, SnIPRE (See Materials and Methods for details), which compares protein-coding polymorphism and divergence data using a robust generalized linear mixed model approach [30].

## Results

### Demographic History of Orang-utan

Ten Bornean and Sumatran wild-caught zoo orang-utans of unknown geographic origin (five per population) were previously sequenced to a median read depth of 6–8X as part of the Orang-utan Genome Sequencing Project [22]. Our analyses of these data at the time indicated a population split-time of 0.4 mya, as well as a current Sumatran effective population size four times larger than that of the Bornean population [22]. To put this split time into perspective, it is similar to the estimated time of the human-Neanderthal split [31].

**Figure 1:**
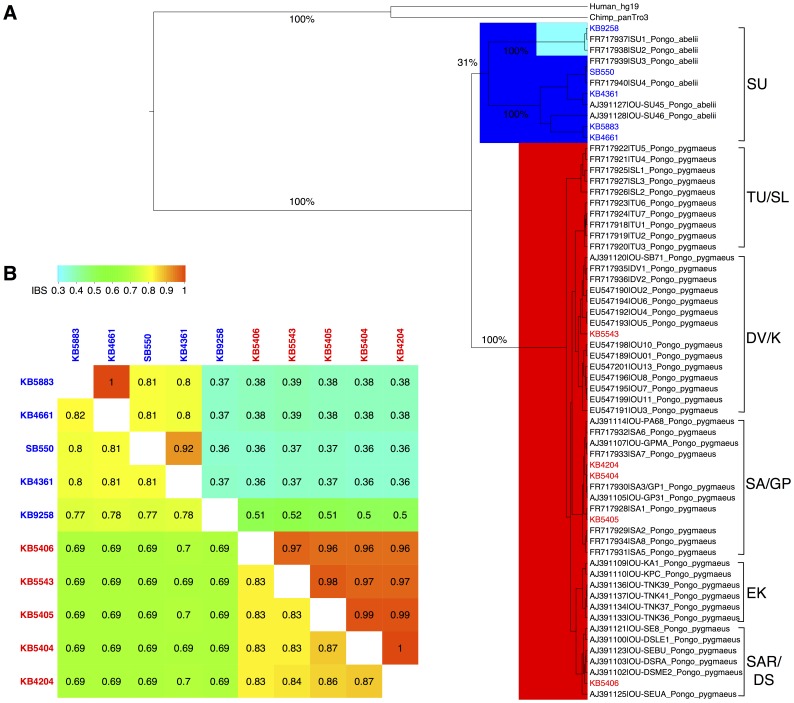
Phylogenetic tree of mtDNA HVRI region and allele sharing matrix of mtDNA and autosomes. (**A**) The phylogenetic tree among human, chimpanzee, Bornean (red) and Sumatran (blue). The nodes shaded in cyan represent the grouping of the three Sumatran individuals that has the furthest distance to the rest clustering of the Sumatran group. The 10 re-sequenced individuals from our study are colored as red (Bornean) or blue (Sumatran). The corresponding geographic origins are shown on the right with the following annotation (SU: Sumatran; TU/SL: Tuanan or Sungai Lading; DV/K: Danum Valley or Kinabatangan; SA/GP: Sabangau or Gunung Palung; EK: East Kalimantan including Kutai national park or Sangatta; SAR/DS: Semongok Wildlife Rehabilitation Centre or Danau Sentarum). (**B**) The IBS allele sharing matrix among the 10 individuals from our study with the same group coloring scheme. The upper and lower triangular matrix represents the IBS sharing of all mtDNA variation loci (n = 1084) and autosomal variation loci (n = 11,866,619), respectively.

In order to further study the demographic history of the populations from which these 10 wild-caught orang-utan individuals derive and to elucidate their evolutionary relationships, we called mtDNA variants for each of the two populations separately. Since mtDNA is inherited solely from the mother, comparison of mtDNA-inferred demographic history to that inferred from autosomal DNA enables inference of sex-biased migration (i.e., migration involving an unequal number of males and females). We incorporated additional Bornean (n = 59) and Sumatran (n = 7) mtDNA hypervariable region I (HVRI) haplotypes [5], [6] and performed mtDNA phylogenetic analysis (See Materials and Methods for details). The phylogenetic tree inferred from this combined dataset (Figure 1A) places our 10 individuals throughout the tree, which indicates that our samples are representative of this larger data set. While the sample donors for the current study were wild caught, their exact geographic origin is unknown. Based on the known sampling locations of the additional samples and the placement of our samples on the phylogenetic tree, we were able to further localize the likely geographic origin of these samples as indicated in Figure 1A.

Using a Bayesian Markov chain Monte Carlo (MCMC) method implemented in BEAST version 1.6.1 [32], we were able to estimate the time to most recent common ancestor (TMRCA) for the mtDNA of the two populations of orang-utans as 3.67 mya. This is consistent with a previous TMRCA estimate of 3.5 mya based on mtDNA data [5]. In contrast to the shallow tree in the Bornean population, there is a deep divergence in the Sumatran population (Figure 1A), which is consistent with the finding that Sumatran individuals have higher genetic diversity levels than their Bornean counterparts and more population-specific variation [22]. Three Sumatran individuals (including the outlier female Sumatran orang-utan KB9258 from the 10 individuals sequenced here) cluster together far from the rest of the Sumatran individuals (with only 31% posterior probability) on the tree (Figure 1A). The split time between this particular branch and the rest of the Sumatran cluster is estimated to be 3.12 mya. While the Maximum Posterior (MP) tree places this clade with the rest of Sumatra, the low posterior support is consistent with the principle component analysis (PCA, Figure S1 in File S1) results placing this clade almost equidistant between the main Sumatra and Borneo clades.

Demographic histories consistent with this phylogeny involve population structure within and gene flow between the two orang-utan populations. A continuum of models ranging from ancient substructure of Sumatran and Bornean orang-utans to complex speciation with on-going gene flow is possible. The first scenario suggests the presence of multiple source populations (with several on the island of Sumatra alone) and very little migration among them, leading to deep coalescent times for alleles sampled from multiple demes [33]. The second model that could produce the observed phylogeny is hybridization between Sumatran and Bornean orang-utan populations post-split. This scenario is consistent with the region’s known history of climatic fluctuations that created land bridges [1], [2] which permitted gene flow among incipient populations, followed by subsequent isolation [4], [5]. Under the second scenario, the outlier female Sumatran orang-utan KB9258 and the other two Sumatran individuals could have descended from a rare female migrant from the Bornean population to the Sumatran population, which caused the Bornean mtDNA type to persist in Sumatra after the populations diverged. Note that a combination of these or other histories may also be consistent with this data. The discrepancy between the nuclear and mtDNA split times is consistent with male-biased migration and semi-solitary social organization of orang-utans.

To corroborate the HVRI phylogenetic tree, we examined allele sharing across the 10 orang-utan individuals by calculating identity by state (IBS) coefficients (i.e., the proportion of times a given pair of individuals have the same genotype across SNPs) among all pairs of individuals for autosomal genomic regions and for the whole mtDNA region, separately (Figure 1B). With the exception of the Sumatran individual KB9258, we find that for both populations, the estimated level of allele sharing is consistent with the population assignments as identified by the PCA analysis (Figure S1 in File S1), which suggests that the majority of genetic variation is found within (and not among) populations. Moreover, the Bornean population has higher IBS autosomal and mtDNA coefficients than the Sumatran population, which is consistent with the higher level of nucleotide diversity observed in Sumatran orang-utans [22] and a larger inferred effective population size.

The IBS coefficients imply that the outlier Sumatran individual (KB9258) has more autosomal allele sharing with other Sumatran individuals but more mtDNA allele sharing with the Bornean population. This result differs from the phylogeny estimated from the HVRI of the mitochondria (Figure 1A) where KB9258 is grouped with the other Sumatran individuals via a long branch with relatively low support.

Zoo records indicate that individual KB9258 was indeed caught in the wild, which rules out the possibility of recent ancestry from Bornean individuals due to interbreeding in captivity. Interestingly, two recent studies by Nater at al. [7], [8] also found an outlier group of Sumatran orang-utans living in Batang Toru (BT), revealed by comparing phylogeographic patterns using both mtDNA and Y-chromosomal data. The location and divergence age of the BT group in their mtDNA phylogenetic tree are very similar to our KB9258 clade in Figure 1. To test the relationship of KB9258 with the BT group, we downloaded the mtDNA sequence data from the BT group and compared with KB9258 and found that the sequence of KB9258 is 100% identical to sample BT4 in the HVRI region. Combined with the results of Nater et al. [7], [8], the most likely explanation for the KB9258 clade is it came from Batang Toru, south of Lake Toba. Lake Toba, formed after a series of volcanic eruptions, serves as a dispersal barrier between BT and the other Sumatran populations on northern side of the lake. BT could be an ancient subpopulation where female philopatry and limited dispersal have contributed to the much larger estimate of mtDNA divergence as compared to the autosomes. Male dispersal across Lake Toba could then facilitate gene flow of autosomes and Y-chromosomes, leading to lower divergence with other Sumatran populations on those chromosomes.

### Natural Selection in Orang-utan

**Figure 2:**
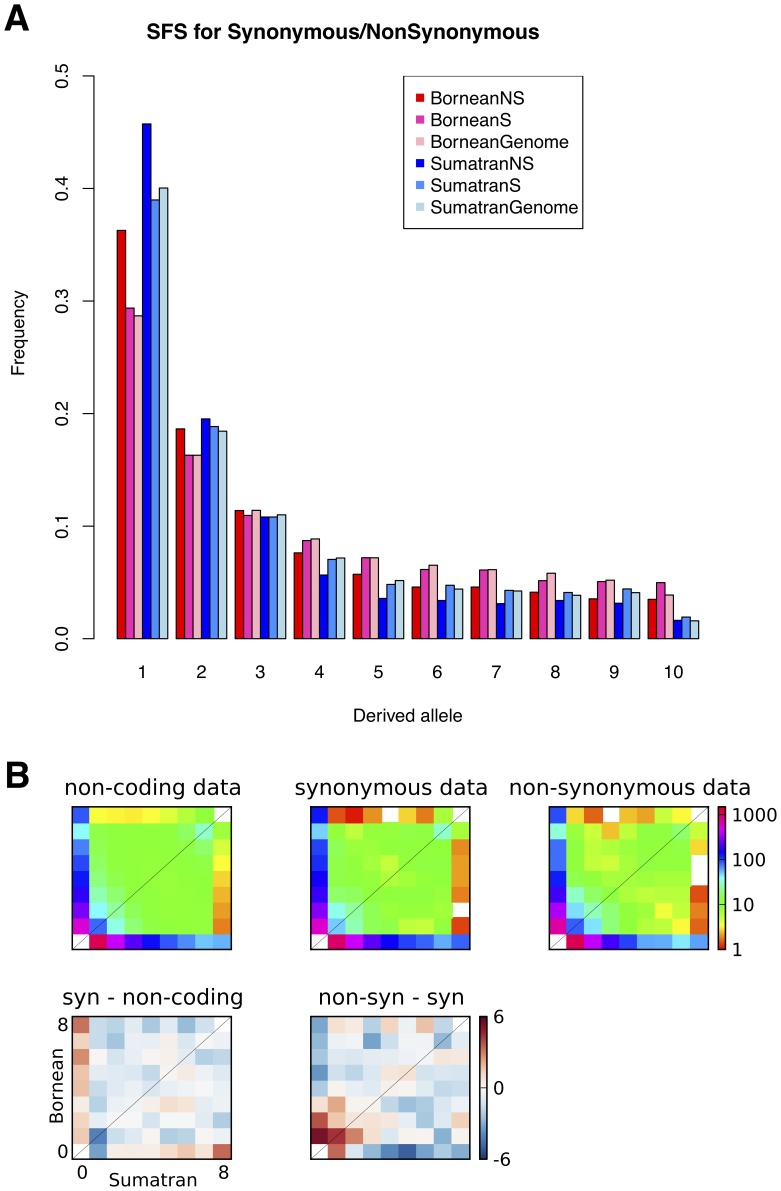
SFS analysis. (**A**) SFS for all genomic SNPs (Genome), synonymous (S) or non-synonymous (NS) SNPs in Bornean (Red) and Sumatran (Blue) population. (**B**) Two-dimensional SFS (Bornean on Y axis and Sumatran on X axis). Top row: Spectra for different functional categories of SNPs. To make all the spectra directly comparable, the non-coding and synonymous spectra have been re-scaled to represent the same number of segregating SNPs as the non-synonymous spectrum. Bottom row: residuals between pairs of spectra. Bottom-left compares synonymous and non-coding spectra, and bottom-right compares non-synonymous and synonymous spectra. Red and blue entries indicate, respectively, that the first spectrum has greater or fewer SNPs in that entry than the second.

We annotated all autosomal SNPs and obtained a total of 20,864 exonic SNPs (12,265 synonymous and 8,600 non-synonymous). The distribution of the SNP counts binned by the derived allele frequency is shown in Figure S2 in File S1. The one-dimensional SFS for the genomic, synonymous and non-synonymous SNPs are shown in Figure 2A, and the two-dimensional spectra are compared in Figure 2B. Based on the first bin in Figure 2A, we observed proportionally more non-synonymous than synonymous singletons in the SFS for both populations. This finding is consistent with either purifying selection reducing the frequency of these potentially deleterious sites or an insufficient time for selection to act on recent, and therefore low frequency mutations [34], [35]. There is also a strong trend for non-synonymous SNPs to be lower in frequency than synonymous (Figure 2B: Bottom-right).

Using the complete gene 1∶1:1 ortholog alignments between human, orang-utan and chimpanzee, we classified fixed differences between orang-utans and humans as well as polymorphic sites within orang-utans as synonymous or non-synonymous. We found 50,177 and 50,141 fixed synonymous differences relative to human over 6.76 M bases of aligned coding region for Bornean and Sumatran orang-utans, respectively (49,971 were shared among all our orang-utans), with a genomic average synonymous substitution rate of 2.90% and 2.89%, respectively (Table 1). We identified 33,701 and 32,901 fixed non-synonymous differences relative to human for an estimated non-synonymous substitution rate of 0.67% and 0.66%, respectively, for each population (27,103 were shared among all our orang-utans). The non-synonymous and synonymous substitution rates with respect to human are very similar in both orang-utan populations because the split time between the two populations is relatively recent compared to their split time to a human outgroup [22]. We also discovered 5,302 synonymous and 4,042 non-synonymous SNPs among the five Bornean individuals yielding 0.31% for the synonymous polymorphism rate and 0.08% for the non-synonymous polymorphism rate. Likewise, 7,076 synonymous and 4,847 non-synonymous SNPs among the five Sumatran individuals gave us a 0.41% synonymous polymorphism rate and a 0.09% for non-synonymous polymorphism rate. For both synonymous and non-synonymous SNPs, the greater estimated Sumatran polymorphism rates are consistent with previous studies finding higher levels of genetic variability in the Sumatran population [22]. The increase in synonymous variation in the Sumatran group as compared to the Bornean group (∼7 k vs. 5.3 k) is most likely due to the difference in effective population sizes (N_e_) of the two populations. This is consistent with the expectation of populations with larger N_e_ having more neutral variation.

**Table 1:** Summary statistics of McDonald-Kreitman table entries for Bornean and Sumatran population using Human (hg19) as outgroup.

Bornean (Modest Population Size Decline)					
	Divergence	Divergence(%)	Polymorphism	Polymorphism(%)	Length(Mb)*
Syn	50177	2.90	5302	0.31	1.73
Non-Syn	33701	0.67	4042	0.08	5.03
Non-Syn/Syn divergence ratio: 22.97%					
Non-Syn/Syn polymorphism ratio: 26.22%					
**Sumatran (6×Population Size Expansion)**					
	**Divergence**	**Divergence(%)**	**Polymorphism**	**Polymorphism(%)**	**Length(Mb)** *
Syn	50011	2.89	7076	0.41	1.73
Non-Syn	33198	0.66	4847	0.09	5.03
Non-Syn/Syn divergence ratio: 22.94%					
Non-Syn/Syn polymorphism ratio: 23.56%					

* This is total aligned length.

Notably, the ratio of non-synonymous to synonymous differences between human and orang-utans (22.95%) is significantly smaller than the ratio of non-synonymous to synonymous polymorphisms within orang-utans (26.22% and 23.56%) in both the Bornean and Sumatran groups, (Bornean: χ^2^ = 32.95, p-value = 9.44e-09; Sumatran: χ^2^ = 4.60, p-value = 0.03) which indicates an excess of amino acid variation in orang-utans relative to divergence [36]. Moreover, the fact that the ratio of non-synonymous to synonymous substitution and non-synonymous to synonymous polymorphism is slightly smaller within the Bornean (0.86) than Sumatran (0.96) populations indicates that there are more deleterious mutations within the Bornean group.

Our estimates of the number of non-synonymous polymorphisms and differences with human may be downwardly biased by the strictness of our three-way alignment and our procedure of excluding codons with even a single uncallable site. However, our estimated dN/dS ratio of roughly 0.22 is consistent with previous analyses [36], suggesting that our results are not strongly biased.

**Figure 3:**
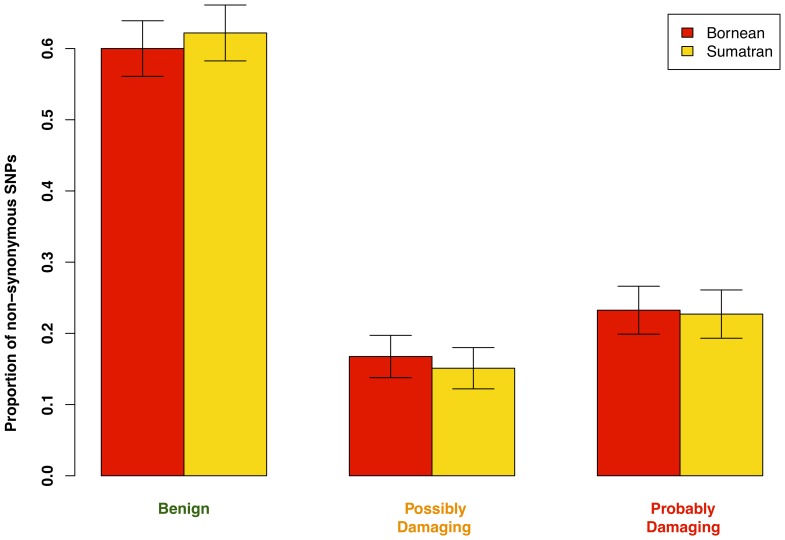
Distribution of the proportion SNPs within each of the PolyPhen categories based on PolyPhen-2 result. Error bars denote 95% confidence intervals on the proportion of SNPs in each category.

To estimate the number of deleterious mutations carried within Bornean and Sumatran groups, we analyzed 2,039 unique predicted transcripts in the orang-utan genome with 1,711 non-synonymous SNPs using PolyPhen-2 [29], which predicts whether the effect of the amino acid change caused by a SNP is benign, possibly damaging or probably damaging based on evolutionary conservation and structural data [37], [38] (see Materials and Methods for details). Comparing the proportion of sites within each of the three categories, there are slightly more benign mutations within the Sumatran group and slightly more possibly damaging and probably damaging mutations within the Bornean group (Figure 3, Table S1 in File S1). The observed trend is not statistically significant but is consistent with the estimated demographic history of a recent bottleneck in Bornean population.

In order to assess the strength of purifying selection within the Bornean and Sumatran populations, we used DaDi [28] to fit the non-synonymous SFS using a model that accounts for both demographic and selective forces. Our baseline demographic model is an isolation-with-migration model in which the two population sizes change size exponentially after divergence [22]. When fitting our selection models to the non-synonymous data, we hold the demographic parameters fixed and set the non-synonymous mutation influx rate θ_non_ to be 2.5 times the influx rate for synonymous mutations θ_syn_ [39]. Given the large size of the non-coding data set fit previously [22], we expect that systematic uncertainties due to model choice to dominate statistical uncertainties in the demographic parameters. Therefore, we evaluated both the full model and two alternative demographic models: a model with no migration, and a model with no population growth (Table S2 in File S1). Both alternative models fit the data much more poorly than the full model, but we find that our inferences about selection are relatively insensitive to the choice of demographic model (Table S3 and S4 in File S1). We have previously observed the same phenomena in other data sets when estimating the distribution of fitness effects (DFE) [39], [40]. Our interpretation is that fitting even a simple demographic model to the frequency spectrum of synonymous sites is often sufficient to allow accurate inference of the DFE on non-synonymous sites, based on the difference between the two frequency spectra.

**Figure 4:**
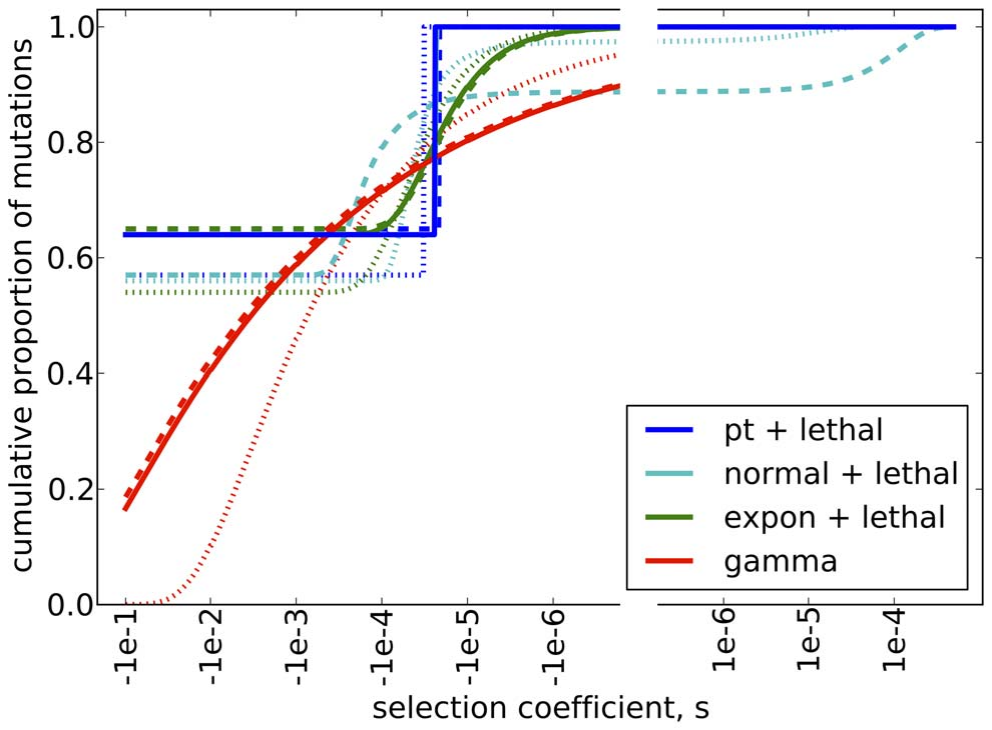
Inferred cumulative distribution of selection coefficients. Solid lines are for the full demographic model, dashed are for the model with no migration, and dotted are for the model with no growth. The break in the axes indicates that the scale is not continuous, because a logarithmic scale cannot span zero.

Fitting a single distribution of selection coefficients to the ancestral, Bornean, and Sumatran populations, we find that the best fitting model is one with 36% of mutations being moderately deleterious, with population-scaled selection coefficient γ = 2N_e_s = −0.85, and the remaining mutations lethal (pt mass + lethal) (Table S3 in File S1). The data are also well fit by a normal distribution of selection coefficients plus a point mass at lethality, an exponential distribution of selection coefficients plus a point mass at lethality, and a gamma distribution of selection coefficients (Table S3 in File S1). All of these distributions point toward roughly 80% of mutations having a selection coefficient more negative than s = −3×10^−5^ (Figure 4, Table 2), and all our inferred distributions for selection coefficients are very similar to the distributions previously inferred for humans [39].

**Table 2:** Percent of mutations within various selective classes for different models of the distribution of selection coefficients and different underlying demographic models.

demographic scenario	selection model	*s*<−10^−2^	−10^−2^< *s*<−10^−3^	−10^−3^< *s*<−10^−4^	−10^−4^< *s*
full model	pt + lethal	64	0	0	36
	expon + lethal	64	0	7	29
	norm + lethal	64	0	0	36
	gamma	47	16	11	26
no migration	pt + lethal	65	0	0	35
	expon + lethal	65	0	5	30
	norm + lethal	57	0	27	16
	gamma	48	16	11	25
no growth	pt + lethal	57	0	0	43
	expon + lethal	54	0	19	27
	norm + lethal	56	0	16	28
	gamma	20	35	21	24

Fitting separate distributions of selection coefficients to the ancestral, Bornean, and Sumatran populations is computationally prohibitive. Fits with fixed selection coefficients plus a point mass at lethality, however, point toward stronger negative selection in Sumatran orang-utans (Table S4 in File S1). These results should be interpreted cautiously, however, because the models also surprisingly point toward γ >0 in the ancestral population, unexpectedly suggesting that ancestral polymorphisms were positively selected.

In order to identify specific loci under positive or negative selection, we applied a statistical mixed model approach called SnIPRE [30]. Using the orang-utan, chimpanzee and human orthologs and filtering genes with fewer than two non-synonymous mutations, we included 2,024 genes in the analysis. Based on the method, many of the genes appear to be neutrally evolving and we identify a large portion of genes (700) under putative negative selection and 23 genes under putative positive selection (Table 3). Functional analysis using the Gene Ontology (GO) database (http://www.geneontology.org/) found the genes under negative selection to be significantly enriched in ion channel activities (Table S5 in File S1) and multiple organismal and system developmental processes including cell communication, signal transduction and protein metabolic process (Table S6 in File S1).

**Table 3:** Genes under positive selection identified by SnIPRE.

Gene under positive selection		
Transcript ID	Description	Gene
knownGene.uc001vjy.1.1	CDNA FLJ35379 fis, clone SKMUS2006481.	AX747676
knownGene.uc002kni.1.1	Homo sapiens cDNA FLJ38028 fis, clone CTONG2013222.	AK095347
knownGene.uc002yvu.1.1	Homo sapiens DSCR6a mRNA, complete cds.	DSCR6a
refGene.NM_000201.1	intercellular adhesion molecule 1 precursor	ICAM1
refGene.NM_002099.1	glycophorin A precursor	GYPA
refGene.NM_002864.1	pregnancy-zone protein	PZP
refGene.NM_004900.1	apolipoprotein B mRNA editing enzyme, catalytic	APOBEC3B
refGene.NM_005397.1	podocalyxin-like precursor isoform 2	PODXL
refGene.NM_005547.1	Involucrin	IVL
refGene.NM_014508.1	apolipoprotein B mRNA editing enzyme, catalytic	APOBEC3C
refGene.NM_016190.1	Cornulin	CRNN
refGene.NM_017716.1	membrane-spanning 4-domains, subfamily A, member	MS4A12
refGene.NM_023922.1	taste receptor, type 2, member 14	TAS2R14
refGene.NM_030972.1	zinc finger protein 611	ZNF611
refGene.NM_033004.1	NLR family, pyrin domain containing 1 isoform 1	NLRP1
refGene.NM_033049.1	mucin 13, epithelial transmembrane	MUC13
refGene.NM_138337.1	myeloid inhibitory C-type lectin-like receptor	CLEC12A
refGene.NM_172241.1	cutaneous T-cell lymphoma-associated antigen 1	CTAGE1
vegaGene.OTTHUMT00000150638.1	carcinoembryonic antigen-related cell adhesion molecule 3	CEACAM3
refGene.NM_001423.1*	epithelial membrane protein 1	EMP1
refGene.NM_001099733.1**	NULL	NULL
refGene.NM_001008743.1**	sulfotransferase family, cytosolic, 1C, member	SULT1C3
knownGene.uc003byh.1.1**	TPRXL protein	TPRXL

* and ** denote genes identified as putative positively selected on orang-utan and human lineage, respectively, in previous study [22].

For the 23 positively selected genes by the SnIPRE criteria, we do not see a clear trend of enrichment of GO terms. The small number of genes that showed signature of positive selection could be due to the estimate from SnIPRE being a shrinkage estimate of the selection effect and therefore requiring stronger evidence in order to detect positive selection, or our stringent requirement for the 1∶1:1 orthologous set to have 90% identity, or a combination of both. Nonetheless, we do find some interesting anti-viral and pathogen related genes. For example, two of the *APOBEC3* family members, *APOBEC3B* and *APOBEC3C* are both adaptively evolving in orang-utans and the human homologs are documented to be potent protectors against simian immuno-deficiency virus (SIV) [41]. Other studies suggest human *APOBEC3B* is a potent inhibitor of HIV-1 infectivity [42]. The *APOBEC* gene family encode enzymes for cytosine-to-uracil editing, which serve as one important defense mechanism against retroviral infection for primates [43]. Several primate molecular evolution studies have suggested that the *APOBEC3* gene family were under strong positive selection in primates [44]–[46]. It is also noteworthy that one recent study of the gorilla genome also showed strong evidence of positive selection on the *APOBEC3* genes [47].

Other pathogen-related loci identified to be under positive selection in orang-utans include glycophorins A (*GYPA*), which is known to be a receptor for multiple viruses including influenza virus [48], Hepatitis A virus [49] and reoviruses [50]. Another positively selected gene is Carcinoembryonic antigen-related cell adhesion molecule 3 (*CEACAM3*), which plays a role in bacterial pathogens binding and invasion to host cells [51]. Similar to humans, orang-utan is vulnerable to SIV and hepatitis [52]. This analysis suggests that orang-utan population might be under selective pressures in fighting viral and bacterial infections in the wild.

Another interesting gene identified to be under positive selection is mucin 13 (*MUC13*), a transmembrane glycoprotein expressed in gastric, colorectal and ovarian cancers [53]. In agreement with previous studies by Achard et al [20], one taste receptor gene *TAS2R14* is also found to be under positive selection, although we cannot rule out the possibility of misalignment because of the existence of many pseudogenes in this family.

## Discussion

The time to the most recent common ancestor of the mtDNA estimated from the combined dataset of our samples and previously published sequences is 3.67 mya. This is consistent with previous estimate of 3.5 mya based on mtDNA variation data [5], but is much older than the population split time estimated from autosomal variation data of 0.4 mya [22]. This difference in times is consistent with sex biased dispersal and the potential reproduction skew since the two populations split [7]. Male-biased dispersal would reduce the coalescent time of the nuclear DNA with every migration event, while the mtDNA will be unaffected because it is maternally inherited. This is consistent with previous demographic inference from autosomal data, which suggested a moderate level of migration in the past [22]. Additionally, we find one deeply divergent female Sumatran sample among the 10 sequenced individuals. This individual likely originated from the Botang Tora region on Sumatra island based on mtDNA analysis. Although the geographic barrier of Lake Toba and the male-biased dispersal and potential reproductive skew can explain the pattern we observed, we cannot rule out the possibility of recent gene flow between Sumatran and Bornean populations. This complex history suggests the need for future study of X and Y chromosome markers in larger samples from both islands for increased insight into the evolutionary history of orang-utans.

Our detailed analysis of the SFS of the Sumatran and Bornean populations stratified by functional classification of SNPs as synonymous or non-synonymous mutations gives insight into how evolutionary forces shaped the observed patterns of polymorphism. The existence of a large proportion of population-specific variants reflects the deep divergence of the two populations. The greater number of SNPs in the Sumatran groups supports the inference of a larger effective population size in that group. The expectation of a population bottleneck leading to the retention of deleterious mutations is supported by a trend of more potentially damaging alleles segregating in the Bornean population. We note that our PolyPhen-2 analysis only includes a limited number of coding variants. PolyPhen-2 only makes predictions for proteins with a homolog in the Uniprot database (http://www.uniprot.org/), and we applied a series of stringent filtering steps which removed any ambiguities or ascertainment biases due to low quality regions of the genome assembly and only kept orang-utan:human homologs with a high level of similarity. Further improvement of filtering steps and the development of PolyPhen-2 models for non-human species may yield a stronger signal of the bottleneck effect.

Previous studies of genes under natural selection in the orang-utan [22] were based on a likelihood ratio test for positive selection using comparative genomic data between species and identified six genes under putative positive selection in the orang-utan lineage. Here we utilized additional information, using both polymorphism within species and divergence between species to infer selection. We find a large proportion of orang-utan genes showed signatures of negative selection, including whole classes of genes related to ion channel activities and multiple organismal and system developmental processes including cell communication, signal transduction, and protein metabolic process. We also find several genes showing signatures of positive selection, which might play an important role in environmental adaption and disease resistance in this species. Our finding of adaptive evolution of the APOBEC3 genes (not seen in orang-utan genome paper [22]) is in agreement with previous studies on primate evolution and corroborates the potential role of the APOBEC gene family in the Alu queiescence in the orang-utans [22]. Notably, among the 23 genes identified as under putative positive selection in our study, only one gene (Table 3) overlapped with positive selected genes on the orang-utan lineage identified in a previous study [22]. The difference in sets of positively selected genes can be attributed to the combination of stringent filtering on genes included in the selection analysis in our study as well as the two selection inference methods used in the two studies. Our study looks for recent selective events using polymorphism and substitution data (SnIPRE) while substitution rate based tests identifies long-term selection acting between species. Thus only genes under both recent and long-term selection would be identified by both methods. Furthermore, three genes (Table 3) showing signatures of positive selection in our study also showed similar selective acting on the human lineage in previous studies [22]. While the finding of few genes under positive selection may reflect the real distribution of fitness effects in orang-utan, we cannot rule out the possibility that it is due to the SnIPRE estimate being a shrinkage estimate of the selection effect. The SnIPRE model estimates the genome-wide distribution of selection effects from the data, and estimates gene specific effects given this distribution. In this application the overwhelming majority of genes appear to have negative gene-specific selection effects. In this context it can be difficult to identify genes with statistically significant positive estimates of selection since our prior information tells us these cases are rare [30].

Using mtDNA and autosomal sequence data, we have performed a detailed analysis of how demographic history and natural selection have shaped orang-utan genomes. Our analysis reveals that the demographic histories of these two closely related populations have played a major role in the evolution of their genomes. We also find that the distribution of selection coefficients inferred for orang-utan population is similar to the distribution previously estimated distribution in humans. When averaged across the entire genome, the vast majority of selective effects are slightly negative, which agrees with the frequent observation that most species show a wide-spread pattern of negative selection with positive selection restricted to particular loci [54]. Our genomic analysis provides useful insights into the evolutionary history of the orang-utan species and the abiotic and biotic factors that may have shaped the evolution of their genomes.

## Materials and Methods

### Nuclear and mtDNA SNP Calling and Analysis

We aligned and filtered Illumina short-read data for 10 individuals sequenced by Locke *et al* as previously described [22]. This yields a median coverage of 6–8×across the autosomal genome and 500–1500×across mtDNA (see Figure S3 in File S1). As part of our involvement in the Orang-utan Genome Consortium, we called 11,866,619 autosomal SNPs [22]. For analyses presented here, we excluded sites with missing data. Mitochondrial SNPs were called at sites with a minimum of 20×coverage for all individuals using a log-likelihood ratio test method developed by us and described previously [55]. We detected 1,090 SNPs among a total of 13,648 callable sites spanning approximately 83% of mtDNA. Sites called as heterozygous within a single sample comprised less than 1% of the total SNP calls. While these variable positions could indicate heteroplasmy, they are most likely caused by false positive SNP calling and were thus discarded from further analysis. The genotype of each of the 10 individuals at each of the callable sites has been submitted to dbSNP (http://www.ncbi.nlm.nih.gov/projects/SNP/snp_viewTable.cgi?type=contact&handle=WUGSC_SNP&batch_id=1054968). Given the high coverage at each site, the estimated false positive (FP) rate and false negative (FN) rate are both negligible and as a result we expect to have discovered the majority of the mutations in the callable mtDNA region with high accuracy. The estimated mtDNA mutation rate corresponds to one SNP every 12.5 bp, which is much higher than the estimated autosomal mutation rate of one SNP every 148.2 bp [22]. A higher mtDNA mutation relative to that of the autosomes is consistent with mutagenic oxidative reduction primarily acting on mtDNA.

To compare our results to previously published mtDNA haplotypes from orang-utans, we focused particular attention on a set of 82 mtDNA SNPs within the hypervariable region I (HVRI). We generated haplotypes for our 10 individuals and combined these with the collapsed HVRI haplotypes from 59 and 7 distinct Bornean and Sumatran haplotypes from previous studies [5], [6]. We constructed a phylogenetic tree of all 76 orang-utan individuals (along with *Homo sapiens* and *Pan troglodytes* as outgroups for fossil calibration) using a Bayesian Markov chain Monte Carlo (MCMC) method implemented in BEAST version 1.6.1 [32]. We tested the adequacy of the burn-in period using Tracer 1.5 (http://beast.bio.ed.ac.uk/Tracer). We obtained an estimate of the TMRCA utilizing two calibration points of *Pan-Homo* divergence and Ponginae-Homininae divergence based on multiple fossils and molecular evidence. We chose a normal prior distribution for the 2 calibration points: *Pan-Homo* divergence with mean of 5.3 mya and standard deviation (SD) of 0.35 mya, spanning a 95% interval of approximately 4.4–6 mya [12], [22], [56], [57]; and *Ponginae-Homininae* divergence with mean of 14 mya and SD of 2 mya, spanning a 95% interval of approximately 10–18 mya [22], [58]–[60]. The wide prior distribution for the second calibration point was chosen to account for the variation of time estimate found in different methods [58], [60]. We used *jModelTest* [61], [62] to estimate parameters of a nucleotide mutation model based on the Akaike information criterion (AIC). The prior for mutation rate was set to follow a Normal distribution with mean of 0.29 with a 95% interval of 0.22 to 0.36 mutations/bp/My for the HVRI region [63].

### Gene Annotations

The gene set we used to define non-synonymous sites consists of the union of all non-overlapping orang-utan genes in the RefSeq, knownGene, and VEGA gene sets (http://compgen.bscb.cornell.edu/~kosiol/orangpsg/datasets/) excluding incomplete genes or genes mapped to chrUn, chrX or unassembled regions of the genome. Out of the potential 8,580 total genes, we further required a minimum of 20% of the coding region to be callable as described previously [22], yielding 7,233 genes considered for further analysis.

### PolyPhen Analysis

All 7,233 predicted transcripts were translated to their corresponding protein sequence based on the high-quality set of orthologous gene groups described above. Gaps in the resulting sequence were removed and N characters were changed to ambiguity codons. We used BLAST to query these protein sequences inferred from the orang-utan transcriptome against the Uniprot database (http://www.uniprot.org). We retained 2,039 uniquely mapping protein sequences with a fully conserved start and stop codon where the sequence identity is at least 90%. We used Polyphen-2 [29] to classify amino acid polymorphisms as having a benign, possibly damaging, or probably damaging effect based on the recommended ternary cutoffs of 0.2 and 0.8.

### Inferring the Distribution of Selection Strength on Coding SNPs

We analyzed the 20,864 exonic SNPs found by the Orang-utan Consortium [22], of which 8,600 were annotated as non-synonymous and 12,264 as synonymous. Genotypes with a likelihood probability ≤0.95 were excluded when constructing the frequency spectra. To utilize sites not called in all individuals, we projected the frequency spectra down to four individuals (eight chromosomes) in each population, which yielded a non-synonymous spectrum with 4,911 segregating SNPs and a synonymous spectrum with 6,938 segregating SNPs. Inferring the ancestral state of these SNPs is difficult because there is no close outgroup to the orang-utan species and sites may have experienced multiple mutations. Thus, to avoid biases caused by misidentification of the ancestral state, our quantitative models were fit using only the folded spectra, which do not require polarization of polymorphisms with respect to the ancestral state.

We estimated the distribution of selection coefficients consistent with the observed non-synonymous frequency spectrum using a modified version of the program DaDi [28] that incorporates the functionality of the program prfreq [39]. The expected SFS was generated based on 2000 values of the scaled selection coefficient (γ = 2 Ns) distributed uniformly on a logarithmic scale between γ = −10^−6^ and γ = −2000, along with γ = 0, and 500 values distributed uniformly on a logarithmic scale from γ = 10^−6^ to γ = 100. Confidence intervals were estimated by bootstrap resampling of the non-synonymous data, which accounts for linkage. This is a conservative approach, because as previously described for humans [39], linkage is not expected to be strong between these sites.

PCA on autosomal SNPs revealed one of the Sumatran individuals (KB9258) to be an outlier (Figure S2 in File S1); PC1 separates Bornean vs. Sumatran individuals and individual KB9258 was placed far from the rest along PC2. To assess the impact of this individual on our inference, all SFS analyses were repeated excluding individual KB9258 and a randomly chosen Bornean individual for each SNP (to maintain symmetry in joint SFS). The resulting eight-by-eight SFS had 40% as many SNPs as when all individuals were included, indicating that many SNPs were private to individual KB9258, consistent with the PCA result. The result of fitting our selection models to the data with KB9258 and the random Bornean individual excluded are shown in the last column of Table S3 and S4 in File S1. This exclusion did not have a significant effect on our results; both the relative fit quality of the models and the best-fit parameter values did not change substantially.

### Inferring Genes under Selection

The 7,233 annotated human-chimp-orang-utan orthologs were filtered to keep only genes with at least two non-synonymous mutations in either the fixed or polymorphic column in a traditional McDonald-Kreitman (MK) table, where polymorphism is defined based on the orang-utan coding SNPs from our data. The resulting 2,024 genes were analyzed using the program SnIPRE (selection inference using Poisson random effects) [30] as follows: each gene was represented by a MK table with the counts of synonymous and non-synonymous polymorphism and divergent SNPs, and a Bayesian Poisson random effects model was used to model the selection effect on each gene. Analyzed genes were classified as being neutral, under negative selection or under positive selection based on the 95% credible interval of the selection coefficient estimate. For those genes identified as being putatively selected, the R package topGO was used to perform a Fisher’s exact test on the Gene Ontology annotations.

## Supporting Information

File S1 **Contains:** ***Figure S1.*** PCA on autosomal SNPs called from the short-read sequencing data for 10 resequenced individuals. ***Figure S2.*** Summary of SFS distribution for raw SNP counts for synonymous and non-synonymous substitutions. ***Figure S3.*** Boxplot of distribution of mtDNA coverages for the 10 previously sequenced individuals. ***Table S1.*** Distribution of SNPs by population and functional class from PolyPhen-2. ***Table S2.*** Parameters for input demographic models. ***Table S3.*** Inferred parameters for single-γ selection models. ***Table S4.*** Inferred parameters for multiple-γ selection models. ***Table S5.*** Enriched GO MF (Molecular Function) categories under negative selection. Only categories with at least 10 genes and Fisher’s exact test P-value <0.05 are listed. ***Table S6.*** Enriched GO BP (Biological Process) categories under negative selection. Only categories with at least 10 genes and Fisher’s exact test P-value <0.05 are listed.
(PDF)
