# Supplementary material for: Population Genomic Analysis Reveals a Rich Speciation and Demographic History of Orang-utans (Pongo pygmaeus and Pongo abelii)
Source: PLoS One. 2013 Oct 23;8(10):e77175. doi: 10.1371/journal.pone.0077175 (PMC3806739; doi:10.1371/journal.pone.0077175)
Supplement: File S1 — Contains: Figure S1. PCA on autosomal SNPs called from the short-read sequencing data for 10 resequenced individuals. Figure S2. Summary of SFS distribution for raw SNP counts for synonymous and non-synonymous substitutions. Figure S3. Boxplot of distribution of mtDNA coverages for the 10 previously sequenced individuals. Table S1. Distribution of SNPs by population and functional class from PolyPhen-2. Table S2. Parameters for input demographic models. Table S3. Inferred parameters for single-γ selection models. Table S4. Inferred parameters for multiple-γ selection models. Table S5. Enriched GO MF (Molecular Function) categories under negative selection. Only categories with at least 10 genes and Fisher’s exact test P-value <0.05 are listed. Table S6. Enriched GO BP (Biological Process) categories under negative selection. Only categories with at least 10 genes and Fisher’s exact test P-value <0.05 are listed. (PDF) [file pone.0077175.s001.pdf]

Population genomic analysis reveals a rich speciation and demographic history of orang-utans (*Pongo pygmaeus and Pongo abelii)*

Xin Ma, Joanna L. Kelley, Kirsten Eilertson, Shaila Musharoff, Jeremiah D. Degenhardt, Andre L. Martins, Tomas Vinar, Carolin Kosiol, Adam Siepel, Ryan N. Gutenkust, Carlos D. Bustamante

Supplementary Figures

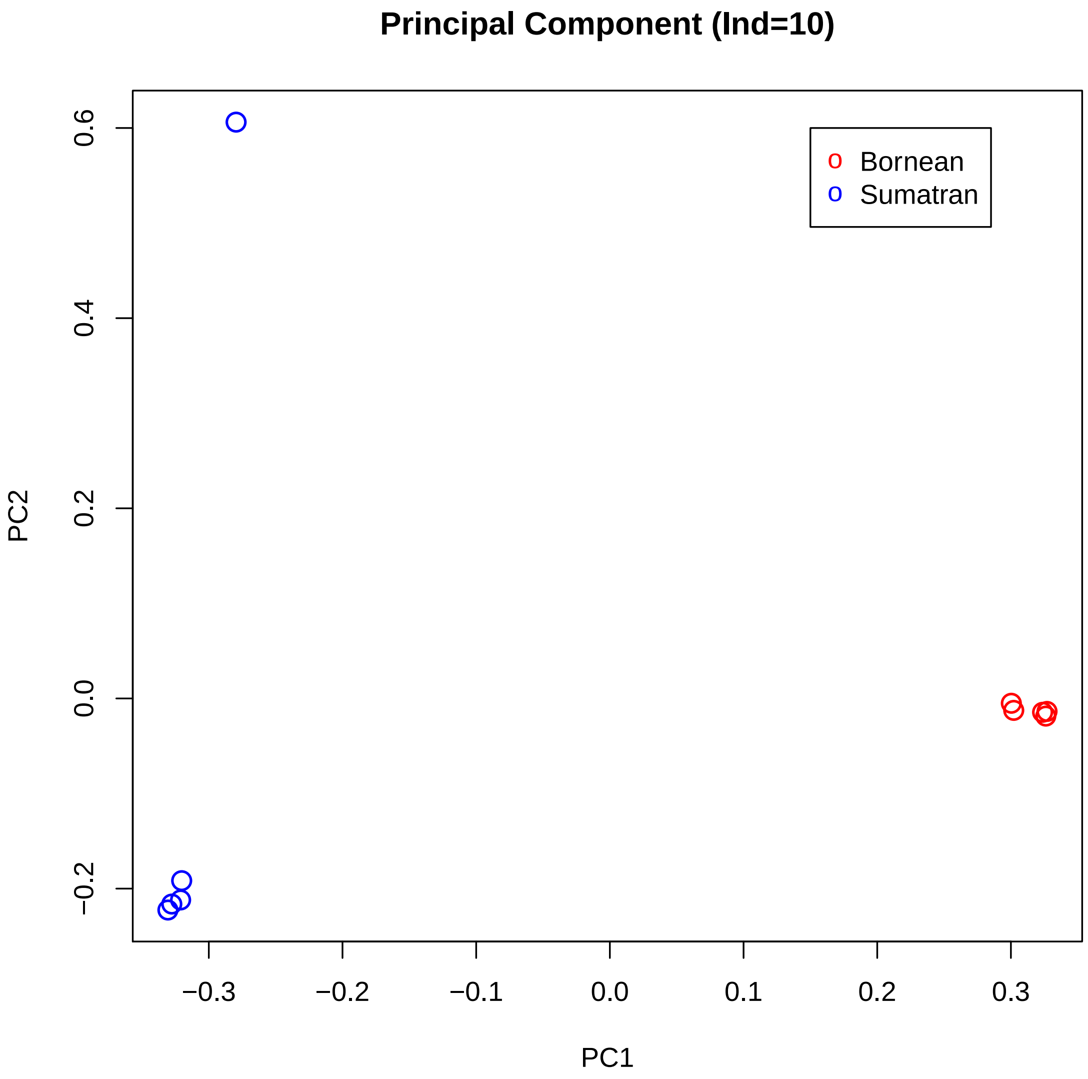

Figure S1: PCA on autosomal SNPs called from the short-read sequencing data for 10 resequenced individuals.

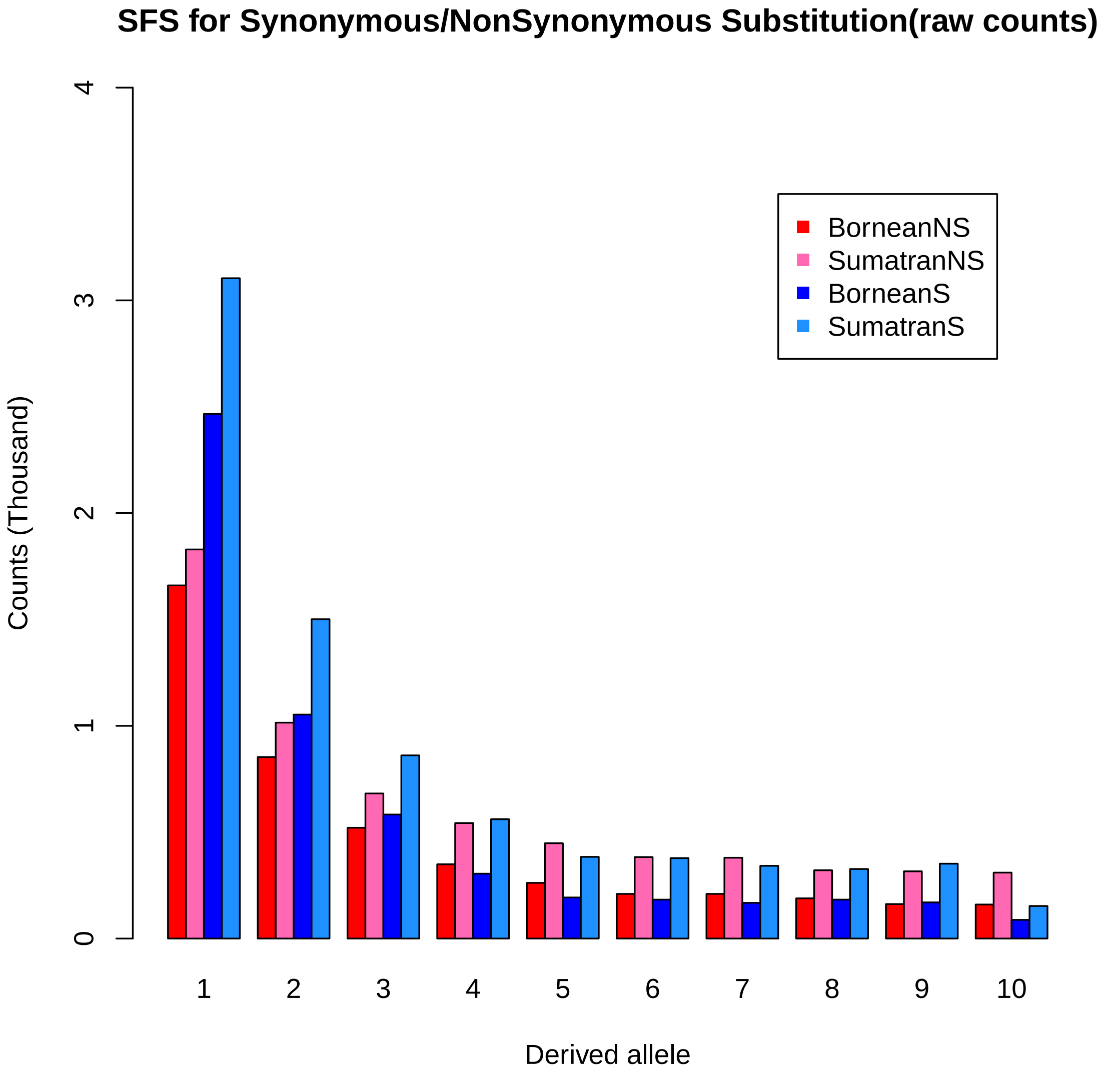

Figure S2: Summary of SFS distribution for raw SNP counts for synonymous and non-synonymous substitutions.

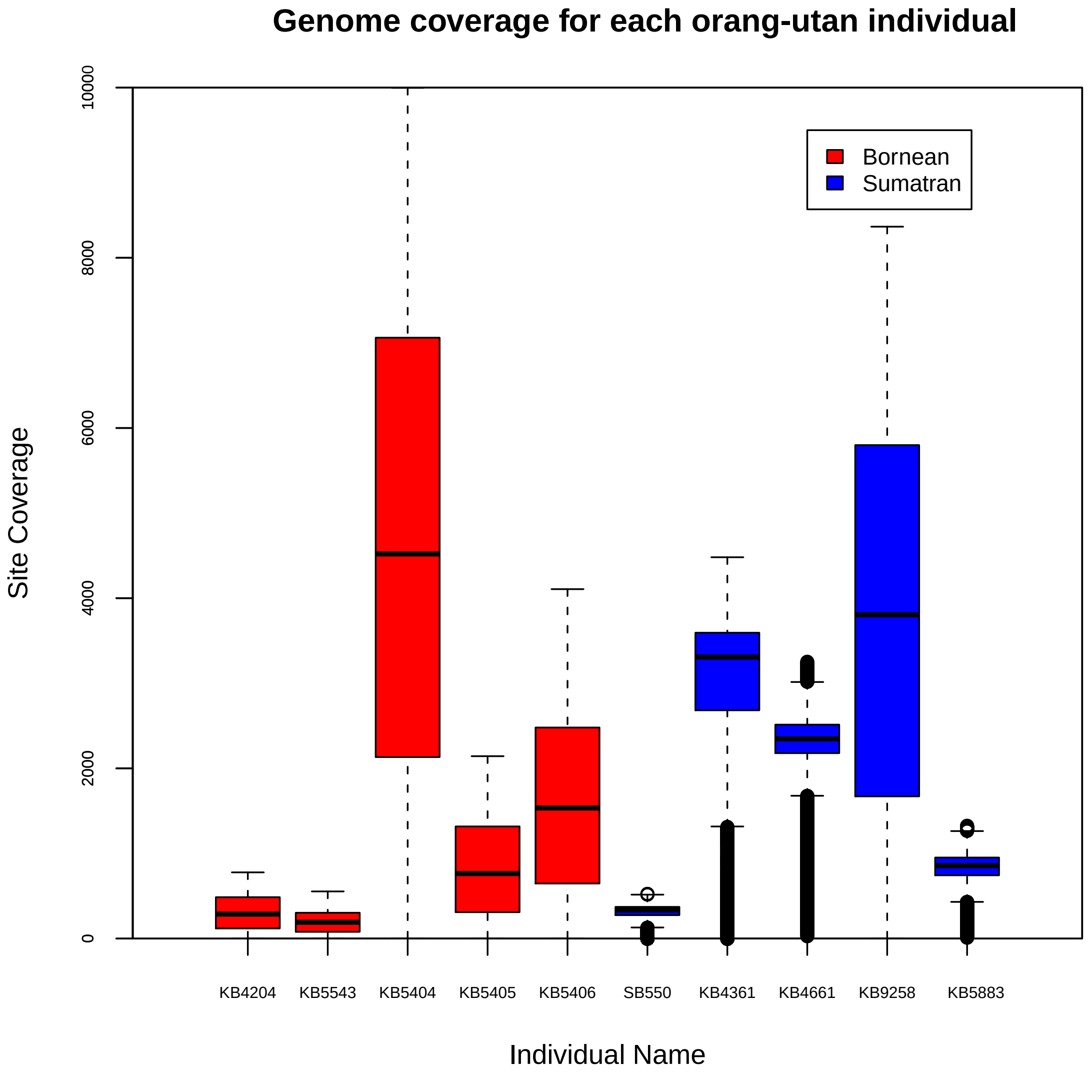

Figure S3: Boxplot of distribution of the mtDNA genome coverages for the 10 resequenced individuals.

Supplementary Tables

Table S1: Distribution of SNPs by population and functional class from PolyPhen-2.

	Benign	Possibly	Probably	Total
Private to Bornean	413	130	155	698
Private to Sumatran	416	108	142	666
Shared	214	45	88	347
Total	1043	283	385	1711

Table S2: Parameters for input demographic models. All three models are variations of the Isolation-with-migration model originally fit to the autosomal data [1]. Parameters are as follows. N_A is the size of the ancestral population. Divergence occurred $2 * N_A * T$ generations in the past, with a fraction s_B of the ancestral population forming the initial Bornean population and a fraction $(1 - s_B)$ forming the initial Sumatran population. The Bornean population then shrank exponentially to a size $\nu_B N_A$ while the Sumatran population grew to a size $\nu_S N_A$. Ongoing migration is such that each generation a fraction $m_{B \leftarrow S}/(2N_A)$ of individuals in the Bornean population were new migrants from the Sumatran population, and vice versa for $m_{S \leftarrow B}$.

model	s_B	ν_B	ν_S	T	$m_{B \leftarrow S}$	$m_{S \leftarrow B}$	N_A	*LL* synonymous	*LL* non-coding
full	0.592	0.491	2.100	0.562	0.395	0.239	17,934	-286.0	-1.62×10^5
no-mig	0.601	0.423	2.389	0.326	0	0	19,445	-330.8	-2.82×10^5
no-growth	0.344	s_B	$1 - s_B$	0.234	0	0	22,359	-369.3	-3.97×10^5

Table S3: Inferred parameters for single-γ selection models

selection model	distribution	param	demographic model full	no-mig	no-growth	no 9528 full
neutral	$\Pr(\gamma = 0) = 1$	LL	-6170.5	-6221.86	-6328.4	-2632.6
fixed (pt mass)	$\Pr(\gamma = k) = 1$	LL	-1396.8	-1510.8	-1073.5	-668.9
		k	-3.1	-3.1	-3.5	-3.1
exponential	$\Pr(\gamma = -x) = \mathrm{EXP}(\lambda)$	LL	-648.7	-739.5	-521.7	-367.9
		λ	10.2	10.6	11.5	10.1
neutral + lethal	$\Pr(\gamma = 0) = p^0; \Pr(\gamma = -\infty) = 1 - p^0$	LL	-313.3	-365.0	-471.6	-216.9
		p^0	0.28	0.28	0.28	0.28
normal	$\Pr(\gamma = x) = \mathrm{NORM}(\mu, \sigma)$	LL	-389.8	-402.9	-318.5	-253.1
		μ	-16.4	-17.2	-19.4	-16.9
		σ	12.0	13.1	14.8	12.5
pt mass + lethal	$\Pr(\gamma = k) = p; \Pr(\gamma = -\infty) = 1 - p$	LL	-257.05	-313.2	-307.9	-195.9
		p	0.36 (0.34,0.37)	0.35	0.43	0.35
		k	-0.85 (-0.99,-0.72)	-0.83	-1.46	-0.82
exponential + lethal	$\Pr(\gamma = -x) = p\,\mathrm{EXP}(\lambda); \Pr(\gamma = -\infty) = 1 - p$	LL	-259.0	-314.3	-307.9	-197.0
		λ	1.05	1.00	2.5	0.96
		p	0.36	0.35	0.46	0.35
exponential + neutral	$\Pr(\gamma = -x) = p\,\mathrm{EXP}(\lambda); \Pr(\gamma = 0) = 1 - p$	LL	-279.9	-326.2	-321.6	-208.7
		λ	126.4	133.7		172.0
		p	0.235	0.233	0.180	0.244
gamma	$\Pr(\gamma = -x) = \mathrm{GAMMA}(\alpha, \beta)$	LL	-268.7	-319.2	-313.0	-202.8
		α	0.16	0.16	0.28	0.15
		β	14392	18725	547	27515
neutral + pt mass + lethal	$\Pr(\gamma = 0) = p^0;$	LL	-257.1	-313.2	-308.0	-195.9
	$\Pr(\gamma = k) = p;$	p^0	0.02	0	0.03	0
	$\Pr(\gamma = -\infty) = 1 - p^0 - p$	p	0.34	0.35	0.40	0.35
		k	-0.91	-0.84	-1.61	-0.81
neutral + gamma	$\Pr(\gamma = 0) = p^0;$	LL	-269.0	-319.3	-313.3	-203.1
	$\Pr(\gamma = -x) = (1 - p^0)\,\mathrm{GAMMA}(\alpha, \beta)$	p^0	0.01	0.01	0.00	0
		α	0.16	0.17	0.31	0.15
		β	13988	13662	316	18285
neutral + exponential + lethal	$\Pr(\gamma = 0) = p^0;$	LL	-259.0	-314.5	-707.9	-197.1
	$\Pr(\gamma = -x) = (1 - p^0 - p)\,\mathrm{EXP}(\lambda);$	p^0	0.00	0.02	0	0
	$\Pr(\gamma = -\infty) = p$	λ	1.08	1.11	2.54	0.99
		p	0.65	0.64	0.54	0.64
normal + lethal	$\Pr(\gamma = x) = p\,\mathrm{NORM}(\mu, \sigma)$	LL	-257.4	-293.2	-307.8	-196.1
	$\Pr(\gamma = -\infty) = 1 - p$	p	0.36	0.43	0.44	0.35
		μ	-0.84	-4.11	-1.81	-0.81
		σ	0.01	6.45	1.15	0.01

Table S4: Inferred parameters for multiple-γ models

selection model	distribution	param	demographic model full	no-mig	no-growth	no 9258 full
3 fixed	$\Pr(\gamma_A = k_A, \gamma_B = k_B, \gamma_S = k_S) = 1$	LL	-836.2			
		k_A	-0.23			
		k_B	-5.64			
		k_S	-6.78			
2 fixed + lethal	$\Pr(\gamma_A = (k_B + k_S)/2, \gamma_B = k_B, \gamma_S = k_S) = p$;	LL	-241.7	-286.4	-279.5	-179.1
	$\Pr(\gamma = -\infty) = 1 - p$	k_B	-0.32	0.03	-0.33	0.00
		k_S	-1.35	-1.69	-2.52	-1.61
		p	0.36	0.36	0.43	0.36
3 fixed + lethal	$\Pr(\gamma_A = k_A, \gamma_B = k_B, \gamma_S = k_S) = p$;	LL	-227.4	-185.6	-168.3	-173.7
	$\Pr(\gamma = -\infty) = 1 - p$	k_A	0.89 (0.40,1.32)	1.29	0.611	1.08
		k_B	-3.97 (-4.57,-2.95)	-8.15	-11.71	-3.91
		k_S	-4.32 (-4.85,-3.51)	-8.43	-12.09	-4.74
		p	0.42 (0.39,0.44)	0.44	0.52	0.42

Table S5: Enriched GO MF (Molecular Function) categories under negative selection. Only categories with at least 10 genes and Fisher's exact test P-value < 0.05 are listed.

GO.ID	Term	Annotated	Significant	Expected	Fisher's P
GO:0005515	protein binding	645	296	255.53	8.6e-6
GO:0001882	nucleoside binding	150	84	59.43	1.4e-5
GO:0001883	purine nucleoside binding	150	84	59.43	1.4e-5
GO:0000166	nucleotide binding	196	105	77.65	1.5e-5
GO:0030554	adenyl nucleotide binding	149	83	59.03	2.1e-5
GO:0008134	transcription factor binding	28	22	11.09	2.6e-5
GO:0003712	transcription cofactor activity	20	17	7.92	3.7e-5
GO:0017076	purine nucleotide binding	177	95	70.12	3.8e-5
GO:0005488	binding	1164	491	461.15	4.1e-5
GO:0005524	ATP binding	136	74	53.88	0.00018
GO:0032559	adenyl ribonucleotide binding	137	74	54.28	0.00024
GO:0003713	transcription coactivator activity	12	11	4.75	0.00027
GO:0032553	ribonucleotide binding	165	86	65.37	0.00038
GO:0032555	purine ribonucleotide binding	165	86	65.37	0.00038
GO:0004714	transmembrane receptor protein tyrosine kinase activity	11	10	4.36	0.00064
GO:0019199	transmembrane receptor protein kinase activity	11	10	4.36	0.00064
GO:0005216	ion channel activity	29	20	11.49	0.00119
GO:0015267	channel activity	29	20	11.49	0.00119
GO:0022803	passive transmembrane transporter activity	29	20	11.49	0.00119
GO:0022838	substrate specific channel activity	29	20	11.49	0.00119
GO:0046873	metal ion transmembrane transporter activity	29	20	11.49	0.00119
GO:0005261	cation channel activity	24	17	9.51	0.00180
GO:0016563	transcription activator activity	24	17	9.51	0.00180
GO:0022836	gated channel activity	26	18	10.3	0.00199
GO:0004672	protein kinase activity	58	34	22.98	0.00220
GO:0003723	RNA binding	52	31	20.6	0.00238
GO:0016301	kinase activity	76	42	30.11	0.00333
GO:0016740	transferase activity	166	82	65.77	0.00425

Continued on next page

Table S5 – continued from previous page

GO.ID	Term	Annotated	Significant	Expected	Fisher's P
GO:0005509	calcium ion binding	109	56	43.18	0.00648
GO:0030695	GTPase regulator activity	40	24	15.85	0.00659
GO:0060589	nucleoside-triphosphatase regulator activity	40	24	15.85	0.00659
GO:0030955	potassium ion binding	13	10	5.15	0.00698
GO:0016773	phosphotransferase activity, alcohol group as acceptor	70	38	27.73	0.00774
GO:0005244	voltage-gated ion channel activity	15	11	5.94	0.00831
GO:0022832	voltage-gated channel activity	15	11	5.94	0.00831
GO:0022843	voltage-gated cation channel activity	15	11	5.94	0.00831
GO:0016772	transferase activity, transferring phosphorus-containing groups	91	47	36.05	0.01096
GO:0022891	substrate-specific transmembrane transporter activity	78	41	30.9	0.01183
GO:0030145	manganese ion binding	12	9	4.75	0.01390
GO:0003824	catalytic activity	505	220	200.07	0.01461
GO:0015075	ion transmembrane transporter activity	68	36	26.94	0.01564
GO:0005249	voltage-gated potassium channel activity	14	10	5.55	0.01583
GO:0005267	potassium channel activity	14	10	5.55	0.01583
GO:0022857	transmembrane transporter activity	86	44	34.07	0.01680
GO:0008237	metallopeptidase activity	18	12	7.13	0.01808
GO:0005083	small GTPase regulator activity	28	17	11.09	0.01848
GO:0008047	enzyme activator activity	28	17	11.09	0.01848
GO:0008324	cation transmembrane transporter activity	54	29	21.39	0.02295
GO:0008233	peptidase activity	52	28	20.6	0.02421
GO:0015276	ligand-gated ion channel activity	15	10	5.94	0.03087
GO:0016564	transcription repressor activity	15	10	5.94	0.03087

Continued on next page

Table S5 – continued from previous page

GO.ID	Term	Annotated	Significant	Expected	Fisher's P
GO:0022834	ligand-gated channel activity	15	10	5.94	0.03087
GO:0004713	protein tyrosine kinase activity	21	13	8.32	0.03149
GO:0031420	alkali metal ion binding	17	11	6.73	0.03155
GO:0005085	guanyl-nucleotide exchange factor activity	19	12	7.53	0.03171
GO:0005096	GTPase activator activity	19	12	7.53	0.03171

Table S6: Enriched GO BP (Biological Process) categories under negative selection. Only categories with at least 10 genes and Fisher's exact test P-value < 0.05 are listed.

GO.ID	Term	Annotated	Significant	Expected	Fisher's P
GO:0048731	system development	158	93	63.94	5.2e-7
GO:0007399	nervous system development	65	45	26.3	1.5e-6
GO:0007275	multicellular organismal development	211	114	85.38	1.0e-5
GO:0048856	anatomical structure development	180	99	72.84	1.6e-5
GO:0007242	intracellular signaling cascade	133	76	53.82	3.2e-5
GO:0044267	cellular protein metabolic process	235	119	95.09	0.00033
GO:0019538	protein metabolic process	241	120	97.52	0.00077
GO:0007167	enzyme linked receptor protein signaling pathway	36	24	14.57	0.00116
GO:0048523	negative regulation of cellular process	110	60	44.51	0.00130
GO:0007517	muscle organ development	13	11	5.26	0.00141
GO:0007165	signal transduction	314	150	127.06	0.00165
GO:0007154	cell communication	347	164	140.42	0.00172
GO:0048519	negative regulation of biological process	120	64	48.56	0.00194
GO:0008361	regulation of cell size	26	18	10.52	0.00262
GO:0006813	potassium ion transport	17	13	6.88	0.00270
GO:0044265	cellular macromolecule catabolic process	28	19	11.33	0.00285
GO:0043687	post-translational protein modification	92	50	37.23	0.00371
GO:0032502	developmental process	300	142	121.4	0.00373
GO:0016051	carbohydrate biosynthetic process	14	11	5.67	0.00417
GO:0019941	modification-dependent protein catabolic process	14	11	5.67	0.00417
GO:0043632	modification-dependent macromolecule catabolic process	14	11	5.67	0.00417
GO:0044257	cellular protein catabolic process	14	11	5.67	0.00417

Continued on next page

Table S6 – continued from previous page

GO.ID	Term	Annotated	Significant	Expected	Fisher's P
GO:0051603	proteolysis involved in cellular protein catabolic process	14	11	5.67	0.00417
GO:0007169	transmembrane receptor protein tyrosine kinase signaling pathway	27	18	10.93	0.00495
GO:0015672	monovalent inorganic cation transport	33	21	13.35	0.00553
GO:0007264	small GTPase mediated signal transduction	45	27	18.21	0.00561
GO:0043170	macromolecule metabolic process	571	254	231.06	0.00571
GO:0032269	negative regulation of cellular protein metabolic process	11	9	4.45	0.00630
GO:0032501	multicellular organismal process	337	156	136.37	0.00717
GO:0010646	regulation of cell communication	56	32	22.66	0.00744
GO:0034962	cellular biopolymer catabolic process	22	15	8.9	0.00756
GO:0009966	regulation of signal transduction	52	30	21.04	0.00788
GO:0016049	cell growth	24	16	9.71	0.00805
GO:0006511	ubiquitin-dependent protein catabolic process	13	10	5.26	0.00831
GO:0034637	cellular carbohydrate biosynthetic process	13	10	5.26	0.00831
GO:0031325	positive regulation of cellular metabolic process	26	17	10.52	0.00844
GO:0051179	localization	277	130	112.09	0.00854
GO:0010604	positive regulation of macromolecule metabolic process	30	19	12.14	0.00890
GO:0009893	positive regulation of metabolic process	32	20	12.95	0.00902
GO:0048513	organ development	115	59	46.54	0.00911
GO:0003012	muscle system process	15	11	6.07	0.00998
GO:0044260	cellular macromolecule metabolic process	556	246	224.99	0.01016
GO:0044248	cellular catabolic process	57	32	23.07	0.01057

Continued on next page

Table S6 – continued from previous page

GO.ID	Term	Annotated	Significant	Expected	Fisher's P
GO:0046467	membrane lipid biosynthetic process	10	8	4.05	0.01303
GO:0001558	regulation of cell growth	21	14	8.5	0.01317
GO:0008285	negative regulation of cell proliferation	23	15	9.31	0.01374
GO:0051056	regulation of small GTPase mediated signal transduction	23	15	9.31	0.01374
GO:0006928	cell motion	39	23	15.78	0.01377
GO:0051674	localization of cell	39	23	15.78	0.01377
GO:0022008	neurogenesis	29	18	11.73	0.01447
GO:0006464	protein modification process	122	61	49.37	0.01614
GO:0051248	negative regulation of protein metabolic process	12	9	4.86	0.01622
GO:0044255	cellular lipid metabolic process	67	36	27.11	0.01678
GO:0006793	phosphorus metabolic process	107	54	43.3	0.01868
GO:0006796	phosphate metabolic process	107	54	43.3	0.01868
GO:0006468	protein amino acid phosphorylation	61	33	24.68	0.01920
GO:0032940	secretion by cell	16	11	6.47	0.02042
GO:0007243	protein kinase cascade	38	22	15.38	0.02092
GO:0016043	cellular component organization	213	100	86.19	0.02176
GO:0045786	negative regulation of cell cycle	20	13	8.09	0.02250
GO:0006811	ion transport	79	41	31.97	0.02262
GO:0040008	regulation of growth	22	14	8.9	0.02299
GO:0044237	cellular metabolic process	701	302	283.66	0.02301
GO:0034960	cellular biopolymer metabolic process	546	239	220.94	0.02321
GO:0043069	negative regulation of programmed cell death	24	15	9.71	0.02322
GO:0060548	negative regulation of cell death	24	15	9.71	0.02322
GO:0032268	regulation of cellular protein metabolic process	26	16	10.52	0.02325
GO:0007155	cell adhesion	88	45	35.61	0.02359

Continued on next page

Table S6 – continued from previous page

GO.ID	Term	Annotated	Significant	Expected	Fisher's P
GO:0022610	biological adhesion	88	45	35.61	0.02359
GO:0051239	regulation of multicellular organismal process	49	27	19.83	0.02484
GO:0008152	metabolic process	749	321	303.09	0.02493
GO:0043412	biopolymer modification	129	63	52.2	0.02657
GO:0009987	cellular process	1130	470	457.26	0.02769
GO:0016310	phosphorylation	91	46	36.82	0.02820
GO:0048522	positive regulation of cellular process	78	40	31.56	0.03041
GO:0006629	lipid metabolic process	87	44	35.2	0.03125
GO:0030001	metal ion transport	37	21	14.97	0.03126
GO:0051093	negative regulation of developmental process	37	21	14.97	0.03126
GO:0006936	muscle contraction	13	9	5.26	0.03402
GO:0007601	visual perception	31	18	12.54	0.03439
GO:0008610	lipid biosynthetic process	31	18	12.54	0.03439
GO:0050953	sensory perception of light stimulus	31	18	12.54	0.03439
GO:0043283	biopolymer metabolic process	555	241	224.58	0.03583
GO:0005976	polysaccharide metabolic process	15	10	6.07	0.03596
GO:0016568	chromatin modification	15	10	6.07	0.03596
GO:0048699	generation of neurons	27	16	10.93	0.03616
GO:0048870	cell motility	27	16	10.93	0.03616
GO:0006810	transport	240	110	97.12	0.03648
GO:0051234	establishment of localization	240	110	97.12	0.03648
GO:0010628	positive regulation of gene expression	17	11	6.88	0.03708
GO:0010557	positive regulation of macromolecule biosynthetic process	19	12	7.69	0.03759
GO:0007156	homophilic cell adhesion	21	13	8.5	0.03765
GO:0009891	positive regulation of biosynthetic process	21	13	8.5	0.03765
GO:0008283	cell proliferation	66	34	26.71	0.04122
GO:0042127	regulation of cell proliferation	40	22	16.19	0.04221

Continued on next page

Table S6 – continued from previous page

GO.ID	Term	Annotated	Significant	Expected	Fisher's P
GO:0044262	cellular carbohydrate metabolic process	40	22	16.19	0.04221
GO:0048468	cell development	40	22	16.19	0.04221
GO:0044238	primary metabolic process	686	293	277.59	0.04791

References

[1] D. P. Locke, L. W. Hillier, W. C. Warren, K. C. Worley, L. V. Nazareth, D. M. Muzny, S. P. Yang, Z. Wang, A. T. Chinwalla, P. Minx, M. Mitreva, L. Cook, K. D. Delehaunty, C. Fronick, H. Schmidt, L. A. Fulton, R. S. Fulton, J. O. Nelson, V. Magrini, C. Pohl, T. A. Graves, C. Markovic, A. Cree, H. H. Dinh, J. Hume, C. L. Kovar, G. R. Fowler, G. Lunter, S. Meader, A. Heger, C. P. Ponting, T. Marques-Bonet, C. Alkan, L. Chen, Z. Cheng, J. M. Kidd, E. E. Eichler, S. White, S. Searle, A. J. Vilella, Y. Chen, P. Flicek, J. Ma, B. Raney, B. Suh, R. Burhans, J. Herrero, D. Haussler, R. Faria, O. Fernando, F. Darre, D. Farre, E. Gazave, M. Oliva, A. Navarro, R. Roberto, O. Capozzi, N. Archidiacono, G. Della Valle, S. Purgato, M. Rocchi, M. K. Konkel, J. A. Walker, B. Ullmer, M. A. Batzer, A. F. Smit, R. Hubley, C. Casola, D. R. Schrider, M. W. Hahn, V. Quesada, X. S. Puente, G. R. Ordonez, C. Lopez-Otin, T. Vinar, B. Brejova, A. Ratan, R. S. Harris, W. Miller, C. Kosiol, H. A. Lawson, V. Taliwal, A. L. Martins, A. Siepel, A. Roychoudhury, X. Ma, J. Degenhardt, C. D. Bustamante, R. N. Gutenkunst, T. Mailund, J. Y. Dutheil, A. Hobolth, M. H. Schierup, O. A. Ryder, Y. Yoshinaga, P. J. de Jong, G. M. Weinstock, J. Rogers, E. R. Mardis, R. A. Gibbs, et al. Comparative and demographic analysis of orang-utan genomes. *Nature*, 469(7331):529–33, 2011.
